# An Investigation of the Pediatric Rigid Bronchoscopy Complication with Three Different Anesthesia Regimes

**DOI:** 10.5812/aapm-150953

**Published:** 2024-09-28

**Authors:** Tahereh Chavoshi, Faranak Rokhtabnak, Nasrin Nouri, Seyedbabak Mojaveraghili, Alireza Eshghi, Reza Salehi

**Affiliations:** 1 Department of Anesthesiology, Aliasghar Children's Hospital, School of Medicine, Iran University of Medical Sciences, Tehran, Iran; 2 Department of Anesthesiology, Firoozgar Hospital, School of Medicine, Iran University of Medical Sciences, Tehran, Iran; 3 Department of Anesthesiology, Rasoul Akram Hospital, School of Medicine, Iran University of Medical Sciences, Tehran, Iran; 4 Department of Anesthesiology and Intensive Care, Faculty of Medicine, Golestan University of Medical Sciences, Gorgan, Iran; 5 Department of Pediatric Pulmonology, Aliasghar Children's Hospital, School of Medicine, Iran University of Medical Sciences, Tehran, Iran

**Keywords:** Rigid Bronchoscopy, Pediatric Anesthesia, Atracurium, Rocuronium, Foreign Body Aspiration, Spontaneous Ventilation, Controlled Ventilation

## Abstract

**Background:**

Foreign body aspiration is common in children and poses a significant risk of morbidity and mortality. Rigid bronchoscopy is the most common method for removing aspirated foreign bodies.

**Objectives:**

Anesthesiologists play a critical role in managing these procedures, aiming to find the best strategies with the fewest complications. This study aims to compare anesthesia-related complications during rigid bronchoscopy in children using muscle relaxants versus no muscle relaxants.

**Methods:**

In this clinical trial, 60 eligible children were randomly divided into three equal groups: SP: Spontaneous ventilation with sevoflurane and propofol; VA: Controlled ventilation with sevoflurane and atracurium; VR: Controlled ventilation with sevoflurane and rocuronium. At the end of anesthesia, complications such as cough, bucking, hypoxemia, laryngospasm, and bronchospasm were compared, along with the pulmonologist’s level of satisfaction, surgery duration, and total anesthesia time in the three groups.

**Results:**

The comparison between the SP, VR, and VA groups revealed the following: No significant difference was found in the incidence of cough and respiratory distress following foreign body aspiration among the three groups (P = 0.262 and P = 0.762, respectively); minimum oxygen saturation during rigid bronchoscopy differed significantly between the groups (P = 0.013); bucking during bronchoscopy was significantly more frequent in the SP group (P = 0.017); laryngospasm was significantly more common in the SP group compared to the other two groups (P = 0.004); agitation during recovery was significantly lower in the propofol (SP) group; pulmonologist satisfaction was highest in the VR group, followed by the VA group, with a significant difference compared to the SP group (P = 0.021); although the SP group experienced more frequent hypoxemia, the difference was not statistically significant; there was no significant difference in anesthesia or bronchoscopy duration across the three groups.

**Conclusions:**

The study results suggest that using muscle relaxants in rigid bronchoscopy offers several advantages, including fewer intraoperative complications such as bucking and laryngospasm. Additionally, controlled ventilation reduced the need for intravenous anesthetics and opioids, minimizing adverse effects and shortening recovery times.

## 1. Background

Aspiration of foreign bodies in children can result in both acute and chronic health issues, necessitating early diagnosis and intervention (1, 2). It is a life-threatening emergency that can lead to cardiorespiratory arrest and sudden death (3, 4). Aspiration is the most common cause of accidental death in children under one year of age. Foreign bodies that migrate to distal airways and are diagnosed late can cause recurrent bronchopneumonia, atelectasis, bronchiectasis, lung abscesses, and fatal airway obstruction (5-7). The gold standard for detecting a foreign body is bronchoscopy, with removal typically performed via rigid bronchoscopy—a long metal instrument of various diameters through which tools can pass to grasp and extract the foreign object. General anesthesia is the preferred method for this procedure (8).

Bronchoscopic intervention for foreign body removal is a high-risk procedure and presents a significant challenge for anesthesiologists. It requires ensuring adequate gas exchange while allowing proper access to the airway for the attending physician during anesthesia (9-11). There is ongoing debate regarding the optimal ventilation method for anesthesia in these cases: Spontaneous versus controlled ventilation. Some anesthesiologists advocate for maintaining spontaneous breathing, arguing that this reduces the risk of dislodging the foreign body, which could otherwise lead to complete airway obstruction (12-14). Additionally, despite the non-parallel resistance caused by the presence of the foreign body, gas exchange may be better preserved with spontaneous ventilation.

However, other anesthesiologists prefer the use of muscle relaxants and controlled ventilation. They believe that this method minimizes airway complications such as coughing, laryngospasm, bronchospasm, and tracheal rupture during bronchoscopy. It also offers advantages such as preventing patient movement, deepening anesthesia, and facilitating smoother passage of the bronchoscope through the vocal cords. Nevertheless, controlled ventilation also has its drawbacks, including the need for positive pressure ventilation and the potential for the foreign object to be displaced to a more distal location (15-19).

In the method of administering anesthesia while maintaining spontaneous breathing, various drugs have been used, including inhalational agents such as sevoflurane, intravenous hypnotics such as propofol, and short-acting narcotics (20). For controlled ventilation, considering the brevity of the procedure and the need for the rapid reversal of the muscle relaxant in case of airway obstruction, short-acting muscle relaxants such as succinylcholine, atracurium, and rocuronium have been utilized (21-24). However, the use of succinylcholine has become limited to airway emergencies in recent years due to its numerous dangerous side effects, including the risk of arrhythmia and malignant hyperthermia, particularly in children. Consequently, there has been an increasing trend among anesthesiologists to use short-acting non-depolarizing muscle relaxants (25, 26).

Rocuronium has gained considerable attention in recent years due to its rapid onset and dose-dependent duration of action. It can be reversed quickly with the use of sugammadex, a reversal agent that can restore spontaneous breathing within seconds, making it especially valuable in airway emergencies such as difficult intubation. Given the brief duration of the procedure and the dose-dependent effect of rocuronium, using the minimum effective dose in rigid bronchoscopy minimizes the risk of airway irritation, and the drug's short elimination half-life enhances safety. Sugammadex presents a safer and faster alternative to neostigmine, albeit more expensive (27-30). Atracurium has also been employed in rigid bronchoscopy with satisfactory results, though its longer duration of action compared to the procedure can be problematic. After the foreign body is removed, anesthesia must be maintained until the relaxant's effects subside. Using lower doses of atracurium can mitigate this issue (31, 32).

## 2. Objectives

This study aimed to compare respiratory complications, hemodynamic changes, and the duration of anesthesia in rigid bronchoscopy for foreign body removal among three groups: SP: Spontaneous ventilation with sevoflurane and propofol; VA: Controlled ventilation with sevoflurane and atracurium; and VR: Controlled ventilation with sevoflurane and rocuronium, to determine which method is safer.

## 3. Methods

### 3.1. Study Design

This study was conducted as a randomized, controlled, double-blind clinical trial from May 2023 to June 2024 at Hazrat Ali Asghar Children's Hospital in Tehran. After obtaining the code of ethics (IR.IUMS.FMD.REC.1402.026) and the IRCT code (IRCT20230506058100N1), 60 children aged 1 to 6 years, diagnosed with foreign body aspiration, were included in the study following written informed consent from their legal guardians. To reduce stress for the families, it was explained at the outset that if consent was granted and the patient met the study criteria, they would be selected to participate in the trial.

The inclusion criteria for the study were children aged 1 - 6 years who were referred to Hazrat Ali Asghar Children's Hospital with a confirmed diagnosis of foreign body aspiration. The children needed to exhibit a documented history of aspiration or choking, along with clinical signs and symptoms such as cough, respiratory distress, or diminished lung sounds upon auscultation, and be classified as ASA class 1 or 2 in terms of physical condition.

The exclusion criteria included emergency cases, children with asthma or irritable airways, airway abnormalities, drug sensitivities to any of the medications used in the study, neurological disorders or seizures, kidney disease, cardiovascular disease, neuromuscular disorders, or metabolic disorders.

The sample size was determined to be 20 participants per group, based on the incidence of laryngospasm reported in the study by Mashhadi et al., assuming a type I error of 5% and a type II error of 20%, calculated using G-Power software (17). By applying Equation 1:

Equation 1.

$n_{A}={\kappa n}_{B}$

nB=PA(1-PA)κ+PB(1-PB)z(1-α2)+z(1-β)PA-PB2

1-β=Φz-z1-α2+Φ-z-z1-α2 

$z=\frac{P_{A}-P_{B}}{\sqrt{\frac{P_{A}(1-P_{A})}{n_{A}}+\frac{P_{B}(1-P_{B})}{n_{B}}}}$

Patients were randomly divided into three groups after confirming their eligibility and obtaining written consent. Randomization was performed using a random list generated from the Sealed Envelope website. This list was provided to one of the operating room staff responsible for assigning patients to their respective groups, who then informed the researcher of the group assignments.

Upon entering the operating room, all patients received intravenous access, and 0.08 mg/kg of midazolam was administered to facilitate separation from the parents. After being transferred to the operating table, patients were monitored using pulse oximetry, ECG, and non-invasive blood pressure (NIBP) monitoring.

Each patient received 5 cc/kg of normal saline and 1 μg/kg of fentanyl intravenously. Anesthesia was induced using a mask delivering oxygen at a flow rate of 10 liters per minute with FiO_2_ 100% and 8% sevoflurane gas (Abbott Healthcare Pvt. Ltd). Once the patient lost consciousness, the sevoflurane concentration was reduced to 4%, and the oxygen flow was decreased to 6 liters per minute. After 3 minutes, once the appropriate depth of anesthesia was achieved, a pediatric pulmonologist inserted a fiberoptic bronchoscope through the patient's nose into the airway. Using a mask interface and a flexible bronchoscopic line, 1 cc of 1% lidocaine was sprayed onto the glottis, allowing a one-minute waiting period for it to take effect.

After inserting the flexible bronchoscope (Fujifilm, Japan), the location and nature of the foreign body were assessed. If the foreign body was not located in the upper part of the primary airway, larynx, or hypopharynx, the patient was included in the study.

For the SP group (spontaneous breathing), 4% sevoflurane was maintained, and a propofol infusion (Fresofol 1%, Fresenius Kabi Austria) was initiated at a rate of 50 μg/kg/min. No muscle relaxants were administered, and rigid bronchoscopy was initiated after 3 minutes.

For the VR group (rocuronium group), 4% sevoflurane was continued, and 0.3 mg/kg of rocuronium (DarouPakhsh) was administered. After 3 minutes, bronchoscopy was performed.

For the VA group (atracurium group), 4% sevoflurane was also continued, and 0.3 mg/kg of atracurium (Anecur 50, Aburaihan) was administered. Rigid bronchoscopy (KARL STORZ Germany) commenced 3 minutes later.

In the VR and VA groups, no propofol was injected. Oxygen was supplied via the port on the side of the bronchoscope (T section), and controlled ventilation was provided using an Ambu bag connected to the Mapleson D system.

The pulmonologist was blinded to the anesthesia plan, and the anesthesiologist administered the anesthesia according to the randomization protocol. Vital signs, complications, and any additional interventions were documented by a trained nurse who was unaware of the study’s objectives.

Heart rate (HR), blood oxygen saturation (SpO_2_), and mean arterial pressure (MAP) were recorded before anesthesia induction and every 5 minutes during the procedure. Hypoxemia was recorded if SpO_2_ dropped below 90% for more than 15 seconds or if symptoms were present. In cases of coughing, hoarseness, laryngospasm, or bronchospasm, 1 mg/kg of propofol was administered, and the event was recorded in the questionnaire. If symptoms persisted, a second dose was given. If this was still insufficient, succinylcholine was injected. For procedures lasting longer than 30 minutes, a relaxant dose of 0.2 mg/kg of the same drug administered at the start of the procedure was given, and the time was recorded.

After the operation, drugs were discontinued in the SP group, while in the VR and VA groups, oxygen with a face mask and low-dose sevoflurane (1%) continued until the patient’s spontaneous breathing returned. Sevoflurane was then discontinued, and reversal agents—atropine (0.02 mg/kg) and neostigmine (0.05 mg/kg)—were administered. The pulmonologist's satisfaction was recorded on a scale of 1 to 5, based on the frequency of patient movement, displacement of the foreign body in the airway, and the need to interrupt the procedure due to hypoxemia. The satisfaction ratings were defined as follows: (1) very bad; (2) bad; (3) average; (4) good; (5) excellent.

Demographic data, including age, sex, weight, bronchoscopy duration (from the time the bronchoscope passed through the vocal cords until it was removed), and anesthesia duration (from induction to recovery), were documented.

Unwanted side effects during anesthesia, including patient movement, bucking, bronchospasm (spasmodic contraction of the bronchial smooth muscle causing lower airway closure), and laryngospasm (muscle spasms of the vocal cords causing sudden difficulty ventilating with the mask), were recorded. Hypoxemia (SpO_2_ below 90% for more than 15 seconds), foreign body displacement, HR changes greater than 20% from baseline, pneumothorax, and pneumomediastinum were also noted. The need for succinylcholine and any patients excluded from the study were documented.

Child arousal during the recovery period was evaluated using the Cravero scale, with scores ranging from 1 to 5. Patients scoring 4 or 5 were classified as agitated. The Cravero scale is detailed in Table 1. 

**Table 1.** Cravero Scales

Behavior	Score
**Obtunded with no response to stimulation**	1
**Asleep but responsive to movement or stimulation**	2
**Awake and responsive**	3
**Crying (for > 3 min)**	4
**Thrashing behavior that requires restraint**	5

### 3.2. Outcomes

The primary outcomes of the study included the incidence of hypoxemia, bucking, and laryngospasm. Secondary outcomes comprised the frequency of additional propofol injections, agitation during recovery, variations in heart rate and MAP, pulmonologist satisfaction, and the duration of anesthesia.

### 3.3. Statistical Analysis

After data collection, the information was entered into SPSS software for analysis. Frequency and percentage were used to describe qualitative variables, while mean and standard deviation were applied for quantitative variables. To measure relationships, *t*-tests, ANOVA, and chi-squared tests were employed. Additionally, regression modeling was conducted to control for potential confounding variables.

## 4. Results

**Figure 1.**
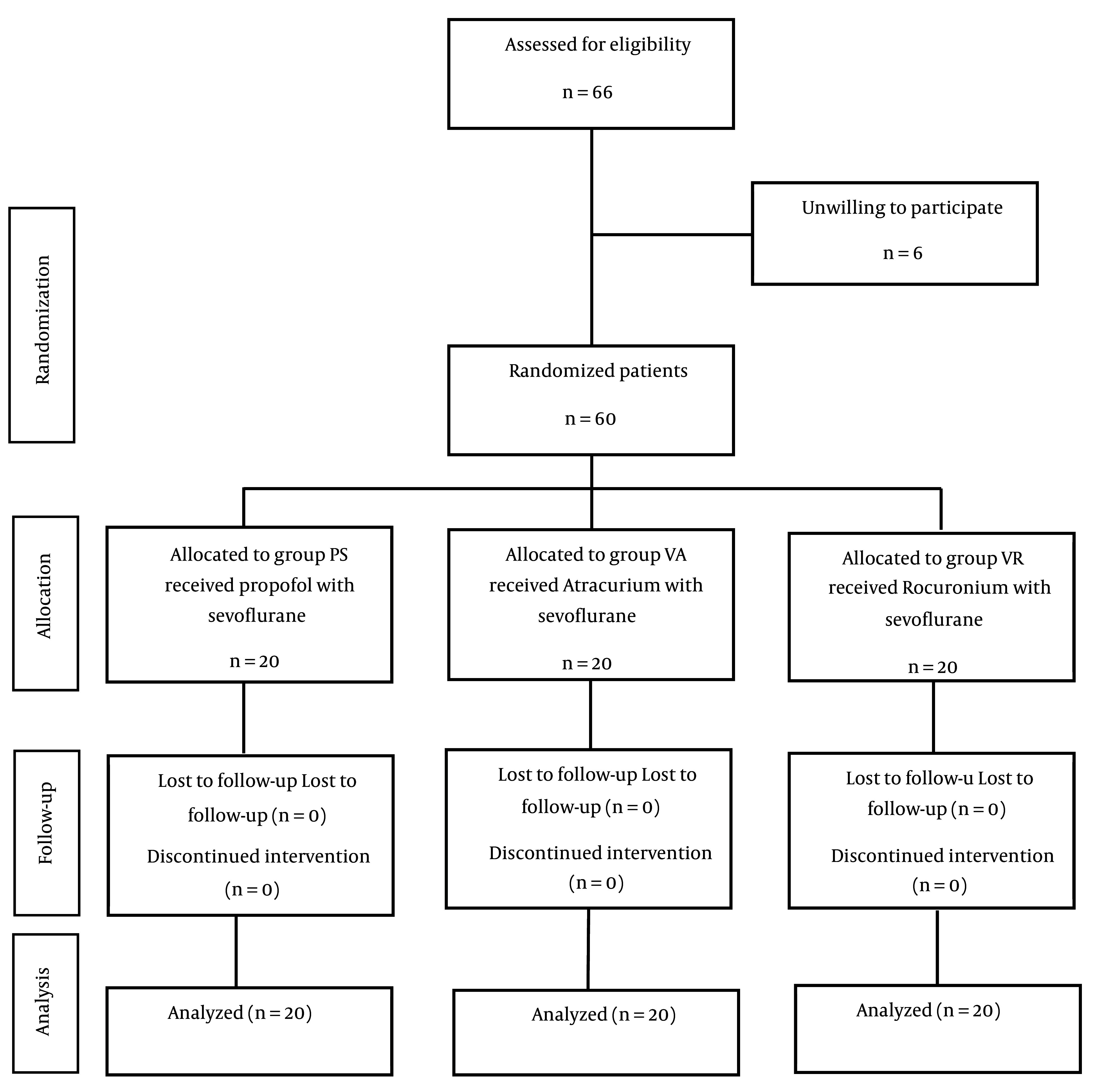
The study flowchart

This study included a total of 60 patients, who were divided into three treatment groups: VA, VR, and SP (Figure 1). There were no significant differences in gender distribution or age among the three groups, with P-values of 0.233 and 0.312, respectively. The demographic characteristics of the patients are presented in Table 2. 

**Table 2.** Demographic Characteristics of Patients ^a, b^

Variable	All patients	VA	VR	SP	P-Value
**Gender**					0.233
Male	36 (60)	13 (65)	14 (70)	9 (45)	
Female	24 (40)	7 (35)	6 (30)	11 (55)	
**Age **	1.99 ± 1.13	1.77 ± 0.89	2.4 ± 1.41	1.8 ± 0.95	0.312
**Weight**	11.65 ± 2.78	10.9 ± 2.29	12.7 ± 3.43	11.35 ± 2.2	0.103

^a^ Values are expressed as No. (%) or mean ± SD.

^b^ SP: Spontaneous ventilation with sevoflurane and propofol; VA: Controlled ventilation with sevoflurane and atracurium; VR: Controlled ventilation with sevoflurane and rocuronium.

Clinical properties prior to anesthesia were compared across the three groups and are presented in Table 3. The results indicate that the interval between aspiration and bronchoscopy did not significantly differ among the three groups (P-value = 0.643). Additionally, the occurrence of cough and respiratory distress prior to anesthesia was similar across all groups (P-values = 0.262 and 0.762, respectively) (Table 3). The majority of aspirated foreign bodies were organic (81.7%), with no significant difference between the groups (P-value = 0.895). The foreign body was predominantly located in the right bronchus (58%), with no significant difference in location between the groups (P-value = 0.880) (Table 3). 

**Table 3.** Comparison of Clinical Specifications Before Anesthesia in Three Groups ^a, b^

Variables	VA	VR	SP	P-Value
**The interval between aspiration and bronchoscopy (day)**	5.58 ± 5.54	5.9 ± 5.2	4.3 ± 3.37	0.643
**Cough (yes)**	19 (95)	20 (100)	20 (100)	0.262
**Respiratory distress (yes)**	8 (40)	8 (40)	10 (50)	0.762
**Aspirated foreign body**				0.895
Organic	17 (85)	16 (80)	16 (80)	
Nonorganic	3 (15)	4 (20)	4 (20)	
**Location of aspirated foreign body**				0.880
Tracheal	1 (5)	2 (10)	1 (5)	
Right bronchus	13 (65)	10 (50)	12 (60)	
Left bronchus	6 (30)	8 (40)	7 (35)	

^a^ Values are expressed as mean ± SD or No. (%).

^b^ SP: Spontaneous ventilation with sevoflurane and propofol; VA: Controlled ventilation with sevoflurane and atracurium; VR: Controlled ventilation with sevoflurane and rocuronium.

The study results indicate that HR, MAP, and oxygen saturation (O_2_sat) before anesthesia were similar across all groups. However, HR at 5 minutes post-anesthesia induction and the minimum and maximum HR during anesthesia showed significant differences between the groups (Table 4). Post hoc analysis revealed that, at 5 minutes after anesthesia, the mean HR difference between the SP and VR groups was -13.9 (95% CI: -23.96, -3.84) and between the SP and VA groups was -14.15 (95% CI: -24.21, -4.09). Minimum HR was also significantly lower in the SP group compared to the VR group (mean difference: -18.4, 95% CI: -28.27, -8.53) and the VA group (mean difference: -18.85, 95% CI: -28.72, -8.98). Similarly, maximum HR was significantly lower in the SP group compared to the VR group (mean difference: -18.5, 95% CI: -28.22, -8.78) and the VA group (mean difference: -17.30, 95% CI: -27.02, -7.58).

**Table 4.** Comparison of Vital Signs During Anesthesia in Three Groups ^a, b^

Vital Signs During Anesthesia	VA	VR	SP	P-Value
**Heart rate before anesthesia**	120.7 ± 16.71	123.3 ± 16.94	122.6 ± 14.8	0.871
**Heart rate 5 minutes after anesthesia**	117.6 ± 21.41	117.35 ± 14.01	103.4 ± 12.2	0.001 ^c^
**Heart rate changes from baseline**	P-value = 0.208	P-value = 0.014	P-value < 0.0001	-
**Minimum heart rate during anesthesia**	109.2 ± 12.97	108.75 ± 13.47	90.35 ± 11.4	< 0.0001 ^c^
**Maximum heart rate during anesthesia**	125.2 ± 11.41	126.4 ± 14.71	107.9 ± 10.9	< 0.0001 ^c^
**MAP before anesthesia**	71.35 ± 8.92	73.7 ± 7.7	72.85 ± 7.8	0.656
**MAP 5 minutes after anesthesia**	65.95 ± 9.1	67.45 ± 6.7	65.55 ± 7.66	0.726
**MAP changes from baseline**	P-value < 0.0001	P-value < 0.0001	P-value < 0.0001	-
**Minimum MAP during anesthesia**	59.9 ± 7.58	63.85 ± 6.73	60.85 ± 5.64	0.160
**Maximum MAP during anesthesia**	70.55 ± 7.98	73.8 ± 6.63	68.9 ± 6.86	0.291
**O**_**2**_ **sat before anesthesia**	96.55 ± 1.46	94.95 ± 3.13	96.35 ± 1.49	0.069
**O**_**2**_ **sat 5 minutes after anesthesia**	99.1 ± 1.21	98.55 ± 1.09	99.35 ± 0.61	0.134
**O**_**2**_ **sat changes from baseline**	P-value < 0.0001	P-value < 0.0001	P-value < 0.0001	-
**Minimum O**_**2**_ **sat during anesthesia**	92.65 ± 6.31	91.8 ± 8.1	85.05 ± 10.7	0.013 ^c^
**Maximum O**_**2**_ **sat during anesthesia**	99.7 ± 0.57	99.25 ± 0.85	99.7 ± 0.57	0.220

^a^ Values are expressed as mean ± SD.

^b^ SP: Spontaneous ventilation with sevoflurane and propofol; VA: Controlled ventilation with sevoflurane and atracurium; VR: Controlled ventilation with sevoflurane and rocuronium.

^c^ Significance at the level < 0.05.

During the procedure, minimum O_2_sat was significantly different among the three groups (P-value = 0.013) (Table 4). Post hoc analysis showed that the mean difference in minimum O_2_sat between the SP and VR groups was -7.1 (95% CI: -13.06, -1.84), and between the SP and VA groups was -6.06 (95% CI: -11.56, -0.39).

Bucking during bronchoscopy was significantly higher in the SP group (P-value = 0.017), although the differences in bucking frequency among the three groups were not statistically significant (P-value = 0.168) (Table 5). Similarly, the incidence of laryngospasm was significantly higher in the SP group compared to the other two groups (P-value = 0.004) (Table 5). 

**Table 5.** Comparison of Clinical Symptoms in Patients During and After Anesthesia Among Three Treatment Groups ^a, b^

Variables	VA	VR	SP	P-Value
**Cough during fiberoptic bronchoscopy (yes)**	7 (35)	9 (45)	9 (45)	0.760
**Frequency of coughing during fiberoptic**				0.168
1 time	5 (71.4)	6 (66.7)	9 (100)	
2 time	2 (28.6)	3 (33.3)	0 (0)	
**Bucking during bronchoscopy (yes)**	3 (15)	1 (5)	8 (40)	0.017 ^c^
**Frequency of bucking**				0.168
1 time	3 (100)	1 (100)	5 (62.5)	
≥ 2 time	0 (0)	0 (0)	3 (37.5)	
**Laryngospasm during bronchoscopy (yes)**	0 (0)	0 (0)	5 (25)	0.004 ^c^
**Bronchospasm during bronchoscopy (yes)**	0 (0)	0 (0)	1 (5)	0.362
**Hypoxemia during bronchoscopy (yes)**	2 (10)	1 (5)	6 (30)	0.064
**Frequency of hypoxemia**				0.062
1 time	2 (100)	1 (100)	5 (83.3)	
2 time	0 (0)	0 (0)	1 (16.7)	
**Propofol injection**				0.017 ^c^
Yes	3 (15)	1 (5)	8 (40)	
No	17 (85)	19 (95)	12 (60)	
**Frequency of propofol injection**				
1 time	2 (66.7)	1 (100)	5 (62.5)	0.355
2 time	1 (33.3)	0 (0)	3 (37.5)	
**Relaxing repetition times (VR, VA)**	1.6 ± 0.59	1.4 ± 0.68	0.05 ± 0.22	< 0.0001 ^c^
**Relaxant dosage (VR, VA)**	0.43 ± 0.13	0.41 ± 0.13	0.025 ± 0.11	< 0.0001 ^c^
**Duration of anesthesia**	45.0 ± 17.39	40.25 ± 14.91	41.25 ± 17.3	0.636
**Duration of bronchoscopy**	34.5 ± 16.85	29.75 ± 14.18	28.0 ± 15.76	0.402
**Agitation in recovery (yes)**	10 (50)	9 (45)	1 (5)	0.004 ^c^
**Time to reach modified alert score 9**	21.1 ± 7.21	17.5 ± 6.17	19.0 ± 6.99	0.201
**Pulmonologist’s satisfaction scores **	4.8	4.9	4.35	0.021 ^c^

^a^ Values are expressed as No. (%) or mean ± SD.

^b^ SP: Spontaneous ventilation with sevoflurane and propofol; VA: Controlled ventilation with sevoflurane and atracurium; VR: Controlled ventilation with sevoflurane and rocuronium.

^c^ Significance at the level < 0.05.

The frequency of propofol injections was significantly greater in the SP group compared to the other groups (P-value = 0.017). However, the total number of propofol injections did not significantly differ among the groups (P-value = 0.355) (Table 5). Alternatively, agitation during recovery was significantly lower in patients who received propofol.

The pulmonologist's satisfaction scores for the three treatment methods revealed that satisfaction was highest in the VR group, followed closely by the VA group, with the lowest satisfaction reported in the SP group. The difference in satisfaction between the SP group and the other two groups was statistically significant (P-value = 0.021) (Table 5). 

## 5. Discussion

In this study, we used three anesthesia methods—SP (spontaneous ventilation), VA (controlled ventilation with atracurium), and VR (controlled ventilation with rocuronium)—in pediatric rigid bronchoscopy. Each method has its advantages and disadvantages. Our results showed that complications during rigid bronchoscopy were less common in anesthesia methods that used muscle relaxants and controlled ventilation. This finding aligns with similar studies, which will be discussed below.

In a study by Chai et al. in China, they compared total intravenous anesthesia (TIVA) and sevoflurane with spontaneous breathing in 435 children undergoing foreign body removal. They found that maintaining anesthesia with sevoflurane resulted in fewer side effects compared to TIVA with propofol and remifentanil. Specifically, complications such as cough, breath-holding, hypoxemia, body movement, laryngospasm, and bronchospasm were significantly more common in the TIVA group (33). Similarly, in our investigation, respiratory complications such as bucking, laryngospasm, and hypoxemia were more prevalent in the SP group.

A meta-analysis by Liu et al. in China compared spontaneous and controlled breathing during bronchoscopy for foreign body removal. Among 864 patients, they found no significant difference in the incidence of desaturation between the two groups (odds ratio: 0.70; 95% CI: 0.30, 1.63). However, they reported a significantly lower incidence of laryngospasm in the controlled breathing group (OR: 0.27; 95% CI: 0.10, 0.76) (34). Our study reached similar conclusions regarding the reduced incidence of laryngospasm in the controlled ventilation groups.

Several studies have examined the use of non-depolarizing relaxant doses. In research by Ghezel-Ahmadi et al. in Germany, they compared succinylcholine with low-dose rocuronium (0.25 mg/kg) in rigid bronchoscopy. They concluded that low-dose rocuronium provided better patient satisfaction and less postoperative myalgia, while succinylcholine offered better intubation conditions at a lower cost compared to rocuronium and sugammadex (35). In our study, we used a low dose of rocuronium alongside sevoflurane and achieved safe and acceptable outcomes with no notable drug side effects.

Finally, a study by Qiu et al. in China over three years involving 2,886 patients used propofol-remifentanil and rocuronium (0.45 mg/kg) to achieve muscle relaxation for rigid bronchoscopy. They concluded that positive-pressure ventilation is an effective and safe technique for removing foreign bodies in children (22).

In summary, our findings support the use of muscle relaxants and controlled ventilation during rigid bronchoscopy as a safer and more effective approach, reducing complications such as laryngospasm and hypoxemia.

In the study by Mashhadi et al., they compared spontaneous and controlled ventilation in children undergoing rigid bronchoscopy for foreign body aspiration (FBA). In the controlled ventilation group, anesthesia induction was performed with fentanyl, sodium thiopental, and atracurium, and maintenance was achieved with a propofol infusion. In the spontaneous ventilation group, both induction and maintenance were done using sevoflurane. The study concluded that complications were significantly lower in the controlled ventilation group, surgeon satisfaction was notably higher, and oxygen desaturation was more frequent in the spontaneous breathing group (17). These results are consistent with our findings.

In a study conducted by Loreau et al. in France in 2023, the choice of anesthesia method for foreign body removal was examined based on the experience of the anesthesiologist. It was found that 58.3% of novice specialists and 77.1% of experienced specialists preferred spontaneous breathing for rigid bronchoscopy (14). This highlights the lack of consensus on the optimal anesthetic technique in pediatric tracheobronchial foreign body management (36).

The findings of our study, which showed fewer complications and greater pulmonologist satisfaction with controlled ventilation, support the use of short-acting muscle relaxants in pediatric FBA cases requiring rigid bronchoscopy. However, to solidify this conclusion, studies with larger sample sizes are necessary.

### 5.1. Limitations

The primary limitation of this study is the small sample size. Another limitation was the heightened anxiety and concern from parents upon hearing the project title, which was mitigated by providing thorough explanations and reassurances that their child's treatment would not be compromised.

### 5.2. Conclusions

There are clear advantages to using controlled ventilation with atracurium or rocuronium during rigid bronchoscopy compared to spontaneous ventilation. Controlled breathing with muscle relaxants reduces the need for hypnotic drugs such as propofol and opioids and results in fewer complications during bronchoscopy. Utilizing low doses of non-depolarizing agents along with sevoflurane provides effective anesthesia quickly and eliminates the need for succinylcholine. Between the two muscle relaxants, atracurium and rocuronium, we favor rocuronium in cases of airway foreign body removal. Rocuronium offers the benefit of rapid reversal with sugammadex when needed and does not cause histamine release. We recommend the use of controlled ventilation with sevoflurane and rocuronium for children with FBA, though further research and discussion are warranted.

## Data Availability

The data are available upon request from the corresponding author.

## Clinical Trial Registration Code:

IRCT20230506058100N1.
